# The PANACEA Project: a multinational model for digital health literacy in inflammatory bowel disease

**DOI:** 10.1093/crocol/otag055

**Published:** 2026-07-06

**Authors:** Viviana Parra Izquierdo, Beatriz Iade Vergara, Marta Benner Machado, Ginary Orduz Diaz, Carolina Samper, Juan Sebastian Frías-Ordoñez, Jahir Soto Mora, Guillermo Veitia

**Affiliations:** Department of Gastroenterology and Rheumatology, Hospital Internacional de Colombia, Bucaramanga, Colombia; Cellular and Molecular Immunology Group (INMUBO), Universidad del Bosque, Bogota, Colombia; Department of Gastroenterology, Asociación Crohn Colitis Ulcerosa Uruguay (ACCU), Montevideo, Uruguay; Department of Gastroenterology, Pontifícia Universidade Católica do Rio Grande do Sul, Porto Alegre, Brazil; Inflammatory Bowel Disease Unit, Hospital Internacional de Colombia, Bucaramanga, Colombia; Fundación Enfermedad Inflamatoria Intestinal Colombiana (FUNEIICO Foundation), Bogotá, Colombia; Department of Gastroenterology, Hospital Internacional de Colombia, Bucaramanga, Colombia; Department of Epidemiology, Universidad El Bosque, Bogotá, Colombia; Department of Gastroenterology, Hospital Vargas de Caracas, Universidad Central de Venezuela, Caracas, Venezuela

**Keywords:** inflammatory bowel disease, Crohn’s disease, ulcerative colitis, patient education, e-learning, digital health, Latin America, health literacy

## Abstract

**Background:**

Inflammatory bowel disease (IBD) represents an increasing public health challenge in Latin America, where structural inequities and limited access to standardized education contribute to gaps in health literacy. Despite the recognized role of patient education in IBD care, region-wide, structured digital initiatives remain limited.

**Objective:**

To describe the implementation, reach, and educational performance of PANACEA (PANamerican Crohn’s and Colitis Educational Approach), a multinational digital education program for patients with IBD and caregivers in Latin America.

**Methods:**

We conducted a multicenter observational descriptive study evaluating four consecutive PANACEA cohorts delivered between 2024 and 2025 through an asynchronous e-learning platform. Adults with IBD or caregivers voluntarily enrolled and completed standardized pre- and post-module knowledge assessments and satisfaction surveys. Program adherence, baseline characteristics, knowledge change, normalized learning gain, and user satisfaction were analyzed using descriptive statistics.

**Results:**

A total of 1366 participants from 15 Latin American countries completed baseline assessments (mean age 41.4 years; 80% female). Sustained adherence to the platform was 53.1%. Cohorts with intermediate baseline knowledge demonstrated improvements in program-specific knowledge assessments (absolute improvement 8–9 points; normalized gain 0.43–0.52), while cohorts with high baseline scores exhibited ceiling effects. Participants with lower baseline knowledge tended to demonstrate greater relative improvements in knowledge scores. Satisfaction exceeded 90% across content quality, usability, and perceived usefulness.

**Conclusions:**

This observational evaluation demonstrates that PANACEA is a feasible and scalable digital education model for IBD in Latin America, achieving broad regional reach, high user satisfaction, and improvements in program-specific knowledge scores, particularly among participants with lower baseline knowledge levels.

## Graphical abstract

**Graphical Abstract:**
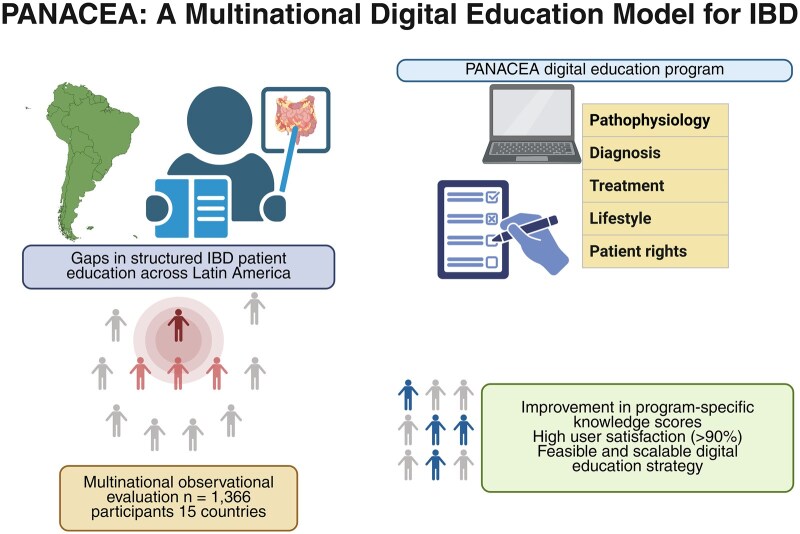

## Introduction

Inflammatory bowel disease (IBD), encompassing ulcerative colitis and Crohn’s disease, poses an increasing challenge to healthcare systems due to its chronic course, impact on quality of life, and need for complex and high-cost therapies. In Latin America, the sustained rise in incidence is compounded by historical inequities in access to specialized care and marked disparities in health literacy. These factors highlight the urgency of strengthening structured educational strategies for patients and caregivers.

Education in IBD transcends the mere transmission of biomedical information—it fosters self-management, treatment adherence, shared decision-making, and empowerment in the face of a fluctuating disease trajectory. Evidence indicates that structured, pedagogically grounded, and culturally adapted educational interventions are associated with improved clinical outcomes and enhanced patient experience.^1^ However, across the region, educational resources remain fragmented, heterogeneous in quality, and poorly aligned with the sociocultural realities of IBD populations.

In this context, digital education has emerged as a strategic tool. Asynchronous e-learning and tele-education platforms overcome geographical and temporal barriers, expand coverage, standardize content, and allow measurable learning analytics. Multiple studies have demonstrated that online education and telemedicine interventions improve disease knowledge, treatment adherence, and quality of life among IBD patients.^2–5^ Nonetheless, large-scale implementation requires addressing persistent challenges, including variable adherence, technological accessibility, and cultural and linguistic diversity.

To respond to these gaps, the Young Group of the Pan American Crohn’s and Colitis Organization (PANCCO) developed PANACEA (*PANamerican Crohn’s And Colitis Educational Approach*), a multinational virtual diploma program designed for patients with IBD and caregivers across Latin America. The initiative provides an accessible, scalable, and culturally relevant educational intervention structured into five thematic modules, each integrating diagnostic, post-learning, and satisfaction assessments.

This study describes the implementation and performance of the PANACEA model within the regional context, evaluating its reach, adherence, knowledge acquisition, and overall satisfaction. Country- and cohort-level differences were also explored, under the hypothesis that participants with lower baseline knowledge would exhibit greater relative knowledge gains.

**Objective:** To describe the implementation of the PANACEA program and to evaluate participation, learning outcomes, and satisfaction among its first four cohorts, with the aim of determining its feasibility, acceptability, and scalability as a digital education model for IBD in Latin America.

## Methods

### Study design and reporting framework

A multicenter, observational, descriptive study was conducted across consecutive educational cohorts with longitudinal pre–post measurements and a final satisfaction survey. The study design and reporting followed the *Strengthening the Reporting of Observational Studies in Epidemiology (STROBE)* guidelines, ensuring methodological transparency and reproducibility across the methods, results, and discussion sections.

### Setting, period, and platform

The PANACEA program was delivered asynchronously through a dedicated e-learning platform accessible via web and mobile interfaces. It was implemented for IBD patients and caregivers across Latin America in four consecutive cohorts between January 2024 and June 2025. All educational content was hosted in a virtual classroom featuring progress tracking, activity logs, and an integrated assessment database.

### Participants

*Inclusion criteria:* (1) adults ≥18 years with a confirmed diagnosis of IBD (ulcerative colitis, Crohn’s disease, or indeterminate colitis) established by their treating physician, or primary caregivers of IBD patients; (2) residence in a Latin American country with internet access; and (3) provision of electronic informed consent and acceptance of data privacy policies.

*Exclusion criteria:* (1) failure to complete the diagnostic pretest of the first module, (2) duplicate registrations (only the first chronologically retained), and (3) voluntary withdrawal prior to initiating module 1.

To ensure data validity and consistency, a rigorous database cleaning process was performed, excluding duplicate entries and incomplete pretests. These measures were methodological adjustments rather than predefined exclusion criteria, aiming to define the final analytical cohort.

### Recruitment strategy

Enrollment was open and voluntary, promoted through patient associations, institutional social media, and the official PANCCO website. No financial incentives were offered.

### Educational intervention: PANACEA

PANACEA comprised five thematic modules: (1) pathophysiology, (2) diagnosis, (3) therapeutic options and safety, (4) lifestyle and self-care (nutrition, mental health, physical activity, sexual and reproductive health), and (5) patient rights and healthcare system navigation.

Each module included:

- Short multimedia lessons (video lectures, readings, and downloadable resources).
- Diagnostic pre-test and post-test (10–20 multiple-choice items).
- A moderated Q&A section with faculty support.

### Content quality and cultural adaptation

Educational materials were developed by a multidisciplinary committee—including gastroenterologists, nurses, nutritionists, psychologists, social workers, and health system managers—and reviewed by patient association representatives to ensure clarity, cultural relevance, and accessibility, using plain language, glossaries, and regionally appropriate Latin American Spanish. A pilot evaluation involving 30 users was conducted prior to program launch to refine usability and content comprehension. Educational modules and knowledge assessments were delivered in Spanish across participating countries, incorporating minor regional linguistic adaptations and contextual examples to facilitate understanding. However, participants’ primary language was not formally recorded, and no standardized cross-cultural linguistic validation of educational or assessment materials was performed.

### Variables and operational definitions

To ensure methodological transparency and reproducibility, all study variables were operationally defined following standardized epidemiological and educational research frameworks. Baseline demographic and clinical variables characterized the study population (Table 1), while adherence and academic performance indicators allowed quantification of engagement and learning outcomes (Table 2). Satisfaction and user experience metrics, collected through validated Likert-scale instruments, provided complementary qualitative insights into program acceptability (Table 3). The explicit definition of each variable and its measurement level facilitated consistent data handling, analytical comparability across cohorts, and robust interpretation of educational effectiveness indicators.

**Table 1:** Baseline characteristics and operational definitions.

Variable	Operational definition	Type of variable
**Age**	Participant’s age in years at the time of enrollment.	Quantitative, continuous
**Sex**	Self-reported gender category: male, female, or unspecified.	Qualitative, nominal
**Country of residence**	Latin American country of residence as reported by the participant.	Qualitative, nominal
**Type of IBD**	Confirmed diagnosis: ulcerative colitis, Crohn’s disease, or indeterminate colitis.	Qualitative, nominal
**Use of biologic therapy**	Current treatment with biologic agents.	Qualitative, dichotomous (yes/no)
**Previous IBD-related surgery**	History of any surgery related to inflammatory bowel disease.	Qualitative, dichotomous (yes/no)
**Current ostomy**	Presence of an ostomy at the time of enrollment.	Qualitative, dichotomous (yes/no)

Abbreviation: IBD = inflammatory bowel disease.

All demographic and clinical variables were self-reported by participants at the time of enrollment through standardized electronic forms.

Data were validated for completeness and consistency prior to analysis.

**Table 2:** Adherence and academic performance variables.

Variable	Operational definition	Type of variable
**Sustained adherence**	Completion of ≥4 out of 5 modules, including both pre- and post-tests for each completed module.	Qualitative, dichotomous (yes/no)
**Module approval**	Post-test score ≥70%.	Qualitative, dichotomous (yes/no)
**Knowledge improvement (Δ)**	Absolute difference between post-test and pretest scores per module and averaged per participant.	Quantitative, continuous
**Normalized gain (g)**	Proportion of the achievable improvement attained: (Post—Pre)/(100—Pre), adjusted for ceiling effect.	Quantitative, continuous

Pretest and post-test refer to the diagnostic and final assessments of each module, respectively.

Normalized gain (g) was calculated using the formula (Post−Pre)/(100−Pre) to correct for potential ceiling effects in high baseline scores.

Sustained adherence was defined a priori as completion of at least 4 of 5 modules, including both evaluations.

**Table 3:** Satisfaction and user experience variables.

Variable	Operational definition	Type of variable
**Content quality**	Five-point Likert scale (1 = very poor, 5 = very good).	Qualitative, ordinal
**Instructor clarity**	Five-point Likert scale (1 = very poor, 5 = very good).	Qualitative, ordinal
**Practical usefulness**	Five-point Likert scale (1 = very low, 5 = very high).	Qualitative, ordinal
**Platform navigation**	Five-point Likert scale (1 = very difficult, 5 = very easy).	Qualitative, ordinal
**Willingness to recommend**	Response to the question “Would you recommend this program?” (yes/no).	Qualitative, dichotomous

Satisfaction metrics were assessed using a 5-point Likert scale (1 = lowest, 5 = highest).

Responses were collected anonymously at program completion to minimize reporting bias.

The “Willingness to recommend” item was analyzed as a dichotomous indicator of overall program acceptability.

### Formulas used

Knowledge improvement (Δ) and normalized gain (g) were calculated as follows:

- Knowledge improvement (Δ): Δ = Post-test−Pre-test
- Normalized gain (g): g = (Post-test−Pre-test)/(100−Pre-test), adjusting for potential ceiling effects.

### Baseline characteristics

Sociodemographic variables included age, sex, and country of residence.

Clinical variables comprised disease type (ulcerative colitis, Crohn’s disease, indeterminate colitis), current use of biologic therapy, prior IBD-related surgery, and presence of a current ostomy.

### Adherence and academic performance

- Sustained adherence: Completion of ≥4 of the 5 program modules and completion of both pre- and post-tests for each module.
- Module approval: Achieving a post-test score ≥70%.

### Satisfaction and user experience

Participant satisfaction was assessed using a 5-point Likert scale for: content quality, instructor clarity, practical usefulness, platform navigation, and willingness to recommend (yes/no).

### Procedures and measurement timeline

1. Platform registration → informed consent → pre-test for Module 1.
2. Module access → post-test upon completion.
3. Sequential repetition for all five modules over 6–8 weeks.
4. Final satisfaction survey at program completion.

Platform logs automatically captured connection time, lesson completion, and test submission.

### Development of knowledge assessments

Knowledge assessments were specifically designed for the PANACEA educational program and aligned with the predefined learning objectives of each module. Item construction was carried out by the multidisciplinary academic committee responsible for curricular development, ensuring content relevance and face validity through expert consensus. Assessments consisted of multiple-choice questions addressing core domains such as disease mechanisms, treatment safety, self-management strategies, lifestyle measures, and navigation of healthcare systems.

Pre-module and post-module assessments followed the same content framework and evaluated identical thematic domains. Although items were conceptually comparable, minor variations in wording and order were introduced to reduce recall bias and improve clarity. This approach aimed to capture knowledge acquisition while minimizing the influence of short-term memorization effects.

Given the implementation-focused and pragmatic nature of the program, formal psychometric validation procedures were not performed. However, internal consistency and comprehensibility were optimized through iterative expert review and pilot testing with representative users prior to program launch.

### Sample size

Given the implementation-focused and descriptive nature of the study, no a priori sample size calculation was performed. All eligible participants meeting the inclusion criteria during the study period were consecutively included in the analysis.

### Data analysis

Continuous variables were summarized as means and ranges (for age). Knowledge improvement (Δ) was computed as the absolute difference between post-test and pretest scores, while normalized gain (g) represented the proportion of potential improvement achieved:


$$g\;=\frac{\mathrm{Post}-\mathrm{Pre}}{100-\mathrm{Pre}},\;\mathrm{adjusting}\;\mathrm{for}\;\mathrm{ceiling}\;\mathrm{effects}.$$

Categorical variables were presented as absolute and relative frequencies.

No hypothesis testing was conducted, as the study followed a descriptive design focused on implementation outcomes. Data normality was not formally tested; however, given the large sample size, normal distribution of mean scores was assumed under the central limit theorem.

### Data management and quality control

Built-in platform validation ensured mandatory completion of critical fields and range checks. Weekly audits were conducted to identify and remove duplicate entries. All data were anonymized before analysis.

### Ethical considerations

All participants provided electronic informed consent prior to enrollment. No personally identifiable information was collected beyond essential sociodemographic variables.

### Anticipated biases and methodological limitations

Potential sources of bias included:

- Self-selection bias, as participants with higher motivation may be more likely to enroll.
- Variable adherence, inherent to virtual environments; an operational adherence threshold was defined and its impact on outcomes analyzed.
- Learning assessment bias, as evaluations were based on internal questionnaires; nevertheless, these instruments were reviewed by a panel of experts and underwent internal consistency analysis.

## Results

### Population and participant flow

A total of 1366 participants were included across four consecutive cohorts (*n* = 602, 288, 209, and 267, respectively). The mean age was 41.4 years (range: 19–85), with a predominance of female participants (80.0%; 1086/1366). Caregivers accounted for 5.3% (72/1366) of enrollees.

### Diagnosis and clinical profile

Reported diagnoses were: ulcerative colitis 62.2% (846/1366), Crohn’s disease 29.5% (409/1366), and indeterminate colitis 2.9% (31/1366).

**Figure 1:**
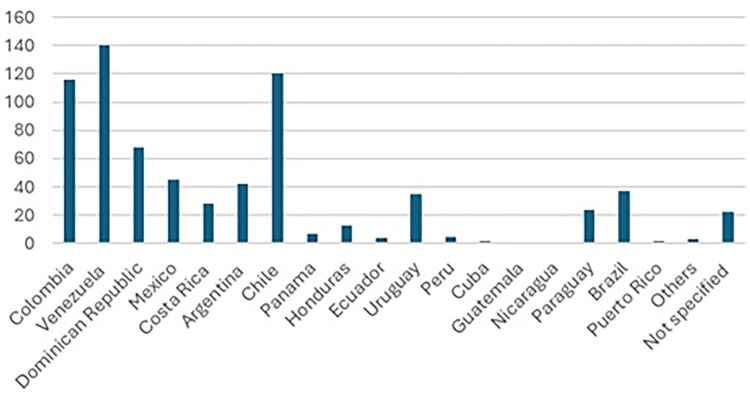
Distribution of participants by country. IBD = inflammatory bowel disease; PANACEA = PANamerican Crohn’s And Colitis Educational Approach.

**Figure 2:**
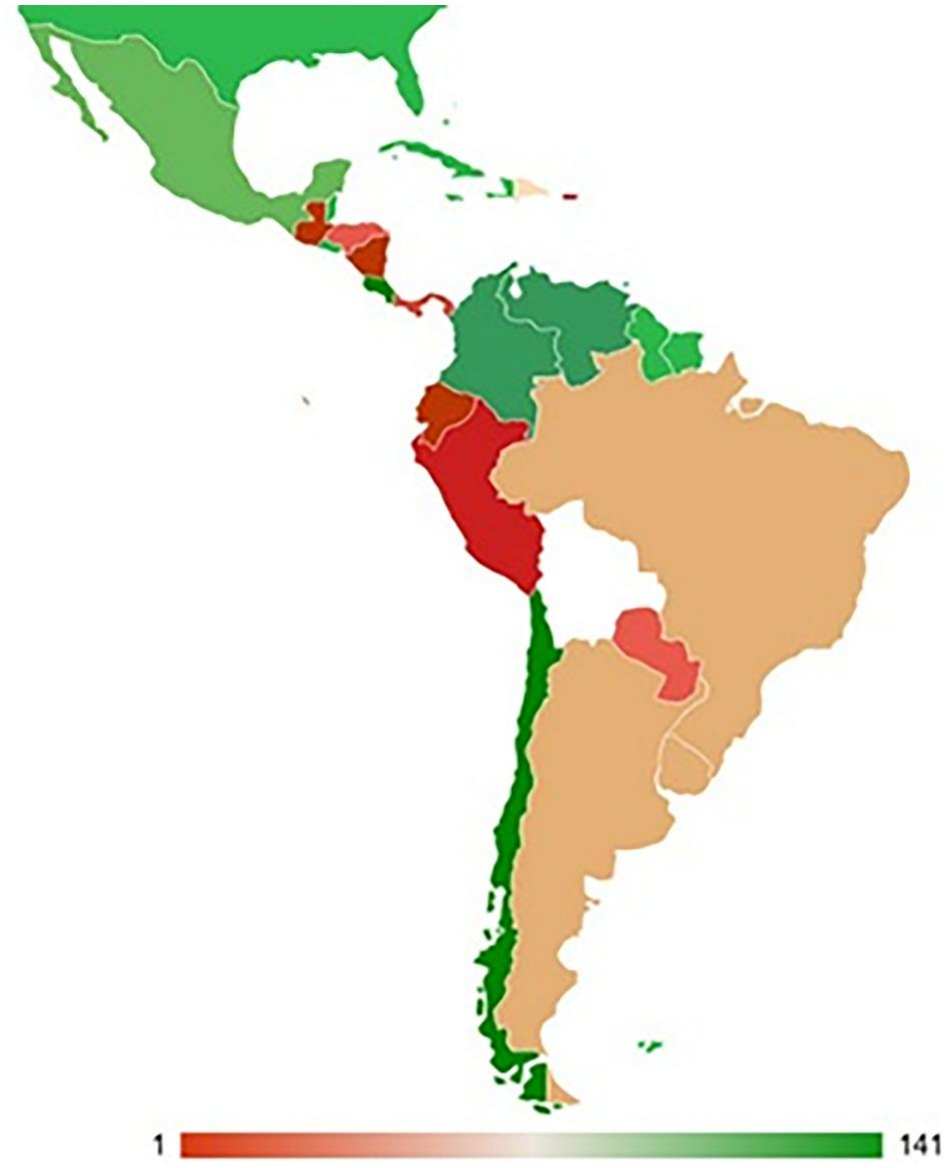
Geographic distribution of participants: Choropleth map illustrating the geographic distribution of participants enrolled in the PANACEA program (*n* = 1366). Shading intensity corresponds to the number of participants per country, with greater intensity indicating higher participation. The map shows broad regional coverage, with the highest concentrations in Colombia, Venezuela, Mexico, Chile, and Argentina, and additional representation from Central American and Caribbean countries. IBD = inflammatory bowel disease; PANACEA = PANamerican Crohn’s And Colitis Educational Approach. Geographic data are presented for illustrative purposes and do not imply national level sampling representativeness. Country boundaries are based on open access geospatial data (Natural Earth dataset).

Regarding treatment history: 45.7% (626/1366) were on biologic therapy, 15.1% (201/1366) had prior IBD-related surgery, and 4.3% (57/1366) had an ostomy at enrollment (see Table 4, Figures 1 and 2).

**Table 4:** Demographic and clinical characteristics of the study population.

Variable	Category	*N*	%
**Age (years)**	Mean (range)	41.4 (19–85)	—
**Sex**	Male	274	20.1
	Female	1086	79.5
	Not reported	6	0.4
**Type of IBD**	Ulcerative colitis	846	62.2
	Crohn’s disease	409	29.5
	Indeterminate colitis	31	2.3
	Uncertain diagnosis	9	0.7
	Caregiver (non-patient)	71	5.3
**Current biologic therapy**	Yes	626	45.7
	No	740	54.3
**History of IBD-related surgery**	Yes	201	15.1
	No	1165	84.9
**Current ostomy**	Yes	57	4.3
	No	1309	95.7
**Total participants**	—	**1366**	**100.0**

Abbreviation: IBD = inflammatory bowel disease.

Percentages are calculated relative to the total number of participants (*n* = 1366).

Age is presented as mean and range (minimum–maximum).

Values may not total 100% due to rounding.

### Adherence and program exposure

Sustained adherence to the platform—defined as completing ≥4/5 modules with both pre- and post-tests—was 53.1% (725/1366). Activity logs revealed linear progression through modules and reduced dropout rates after the third module.

### Academic performance and learning outcomes

Mean pre- and post-test scores per cohort were as follows:

- **Cohort 1:** 88.3% → 87.7% (Δ = –0.6; g = –0.05)—consistent with a ceiling effect.
- **Cohort 2:** 78.9% → 88.0% (Δ = +9.1; g = 0.43)—significant improvement.
- **Cohort 3:** 92.1% → 92.5% (Δ = +0.4; g = 0.05)—ceiling effect.
- **Cohort 4:** 84.3% → 92.4% (Δ = +8.1; g = 0.52)—substantial improvement.

**Figure 3:**
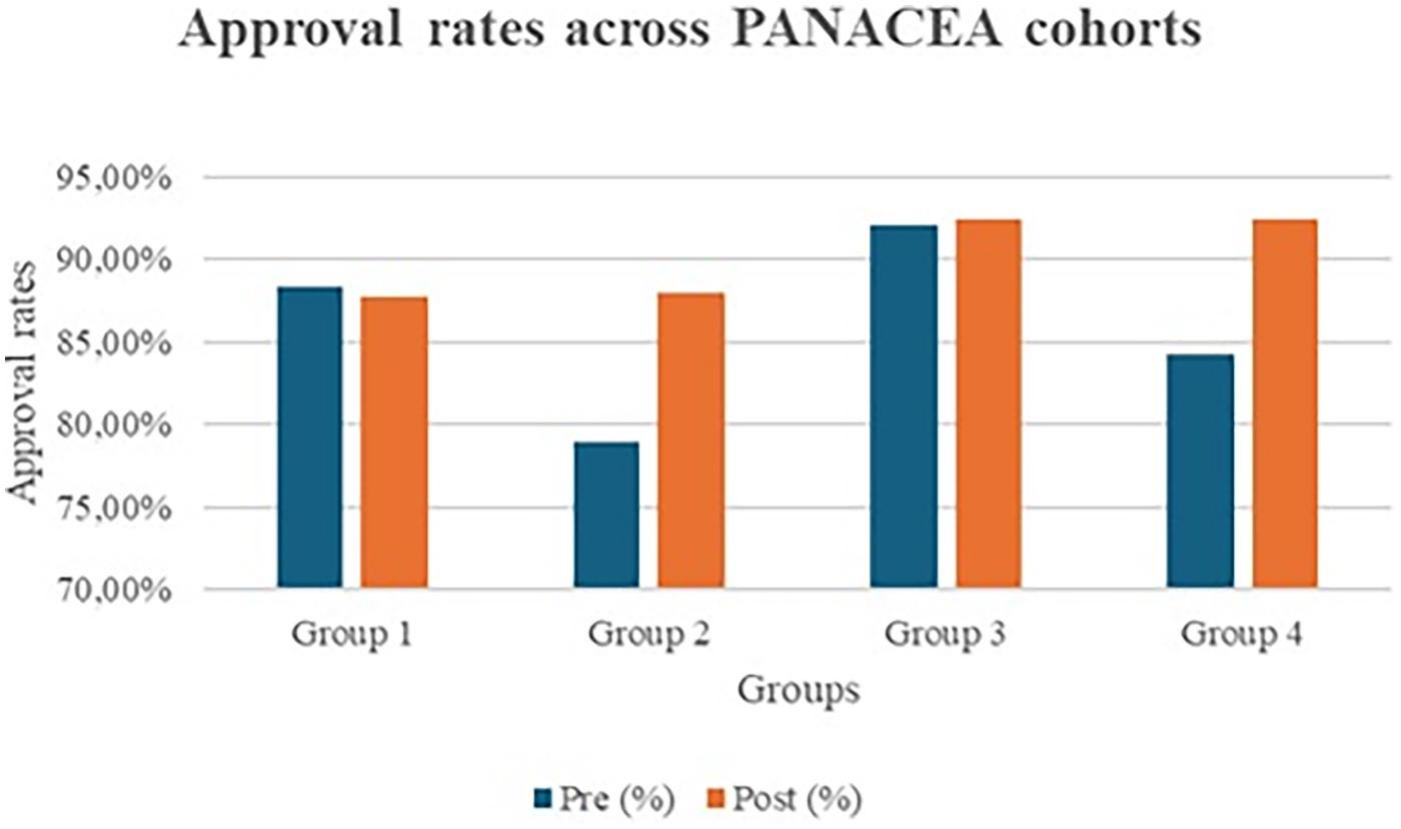
Module approval rates across PANACEA cohorts: Bar chart depicting the percentage of participants achieving module approval (post-test score ≥70%) across the four PANACEA cohorts. Overall approval rates remained consistently high, exceeding 85% in all groups. Cohorts 2 and 4 exhibited the most notable improvement in approval rates after the intervention (78.9% → 88.0% and 84.3% → 92.4%, respectively), while cohorts 1 and 3 maintained stable high performance (>87%), consistent with ceiling effects in knowledge acquisition. Approval rate defined as post-test ≥70%; PANACEA = PANamerican Crohn’s And Colitis Educational Approach. Data represent aggregate approval percentages per cohort (n = 1366 total participants). Variability between cohorts reflects differences in baseline knowledge and prior exposure to structured patient education. The consistently high approval rates across cohorts underscore the pedagogical effectiveness and scalability of the PANACEA e-learning model.

Cohorts with intermediate baseline performance (2 and 4) exhibited absolute improvements of 8–9 points and normalized gains of 43–52%, indicating improvements in knowledge scores within the program-specific assessments. Minimal variation in high-performing cohorts (1 and 3) was consistent with learning saturation (Figure 3).

### Geographical subgroup analysis

Participants originated from 15 Latin American countries (Figure 1). Baseline knowledge scores and learning gains varied across countries. In general, participants from countries with higher baseline scores demonstrated smaller post-intervention improvements, consistent with ceiling effects. Conversely, participants from countries with lower baseline scores tended to show greater absolute and normalized gains following the intervention. These differences suggest heterogeneous baseline exposure to IBD-related information across the region; however, the study was not designed to formally evaluate national educational infrastructures or access to structured IBD education.

### Satisfaction and user experience

Overall satisfaction was high: over 90% rated content and instruction as “very good,” and nearly all participants indicated they would recommend the program.

Qualitative analysis of open-ended responses revealed three recurring themes:

1. Increased confidence in self-management (e.g., understanding treatment and warning signs).
2. Cultural relevance and clarity of language.
3. Practical usefulness for clinical consultations and shared decision-making.

## Discussion

This multinational study describes the implementation of PANACEA, a virtual diploma for IBD patients in Latin America, showing high participation, improvements in program-specific knowledge scores, and sustained satisfaction.

Our findings align with existing evidence showing that well-structured digital interventions can enhance disease knowledge and self-management in IBD populations. Recent randomized trials, such as those using social media–based education, have reported significant gains in disease knowledge and treatment adherence, supporting the efficacy of innovative digital learning modalities.^1^^,^^2^

Similarly, web-based educational portals have yielded sustained improvements in disease-specific domains—such as reproductive health literacy in IBD—lasting beyond six months, indicating the potential durability of cognitive benefits.^3^

Our data also contribute to the growing body of evidence on telemedicine and eHealth in IBD, which consistently demonstrate improvements in quality of life, reduced in-person visits, and high acceptability when educational components are systematically integrated.^4–6^

For example, in the TELE-IBD randomized trial, telemedicine achieved clinical outcomes comparable to standard care while serving as an effective vehicle for patient education and remote follow-up.^5^^,^^6^ Together, these findings underscore the relevance of scalable digital platforms like PANACEA to expand reach, standardize curricula, and mitigate geographic barriers—issues of particular importance in Latin America.

A key insight from this study is the educational gradient observed across cohorts: participants with lower or intermediate baseline knowledge achieved larger absolute and normalized gains, whereas those with high baseline scores exhibited ceiling effects. This pattern is consistent with prior literature on informational needs and learning behaviors in IBD.^7–10^ Although these differences may reflect heterogeneous prior exposure to disease-related information across countries, the present study did not include objective indicators of national educational infrastructure or access to structured IBD education; therefore, these findings should be interpreted as exploratory observations rather than formal comparisons between countries.

Furthermore, validated instruments such as the Crohn’s and Colitis Knowledge Score (CCKNOW) have been shown to capture meaningful differences across educational interventions, reinforcing the need for standardized assessment tools in future program iterations.^11^

The overall adherence rate of 53.1% aligns with prior reports from online learning environments, where completion rates typically decline with program duration. Nonetheless, satisfaction levels remain consistently high, reflecting the acceptability and perceived value of virtual learning in IBD management.^3^^,^^4^^,^^12^ Strategies such as peer mentoring, periodic reminders, simplified infographics, and optional synchronous tutoring could enhance retention without compromising scalability.

While prior systematic reviews acknowledge knowledge improvements after educational interventions, evidence for clinical outcomes (e.g., relapse rates, disease activity) remains heterogeneous due to variable design quality and measurement approaches.^8^ This highlights the need for controlled studies incorporating validated knowledge scales, patient-reported outcomes (PROs), and medium-term clinical endpoints. Additionally, implementation research assessing cost-effectiveness, digital divide, and cultural adaptation is warranted to optimize future scalability.^7–10^^,^^12^

Strengths of our study include the broad regional reach and large sample size, the comprehensive curriculum addressing pathophysiology, diagnosis, therapy, lifestyle, and patient rights, the use of standardized pre- and post-module assessments within the platform, and consistently high levels of participant satisfaction.

Several limitations should be considered when interpreting the findings of this study. First, the non-randomized observational design may have introduced self-selection bias, as individuals with greater motivation or interest in disease education were more likely to participate. Second, knowledge outcomes were measured using program-specific questionnaires developed for educational purposes. Although these instruments were aligned with predefined learning objectives and reviewed by subject-matter experts, they did not undergo formal external psychometric validation. This limits comparability with standardized tools such as the CCKNOW and may affect the precision with which knowledge improvement can be quantified across studies. Third, the absence of longitudinal follow-up precludes assessment of whether improvements in knowledge translated into sustained behavioral changes or measurable clinical outcomes. Additionally, the study did not incorporate objective indicators to characterize national differences in access to structured IBD education or educational infrastructure, which limits the interpretation of geographic variability. Another relevant limitation relates to the linguistic diversity of the participating population. Although educational materials were delivered in regionally adapted Spanish to enhance comprehension, participants’ primary language was not formally recorded and no standardized cross-cultural linguistic validation of educational or assessment content was performed. Consequently, the potential influence of language-related factors on knowledge acquisition could not be formally evaluated. Finally, no formal economic evaluation was conducted, and therefore the cost-effectiveness and broader health system implications of the intervention remain to be determined.

Within these limitations, the PANACEA program appears to represent a feasible strategy to expand access to structured IBD education across diverse settings in Latin America. The observed changes in program-specific knowledge scores and the high levels of participant satisfaction suggest that the platform may serve as a scalable educational resource, particularly in settings where structured educational opportunities are limited. Future iterations of the program will incorporate validated knowledge assessment instruments to allow more precise evaluation of educational outcomes.

## Conclusions

PANACEA represents a feasible and scalable model of digital education for patients with IBD in Latin America, demonstrating broad regional reach, high user satisfaction, and measurable improvements in program-specific knowledge scores, particularly among participants with lower baseline knowledge levels. These findings support the potential role of structured virtual education as a complementary component of patient-centered care, especially in regions with heterogeneous access to specialized educational resources.

Future research should aim to enhance both the methodological rigor and the clinical relevance of digital educational interventions in IBD. The incorporation of validated knowledge assessment instruments, such as the Crohn’s and Colitis Knowledge Score, will be essential to enable standardized evaluation and facilitate comparison across educational programs. Moreover, studies integrating patient-reported outcomes, longitudinal clinical endpoints, and cost-effectiveness analyses are needed to better define the real-world impact and sustainability of such initiatives. Innovative hybrid educational strategies combining asynchronous digital learning with targeted synchronous support may further improve adherence, knowledge retention, and meaningful behavioral translation. Additionally, future program development should include structured linguistic adaptation processes and systematic evaluation of participants’ primary language in order to better understand the influence of cultural and language-related factors on the effectiveness of digital education across diverse populations.

## Data Availability

The datasets generated and analyzed during the current study are not publicly available due to institutional privacy restrictions but are available from the corresponding author upon reasonable request and subject to ethical and data protection approvals.

## Patient and public involvement

Patient representatives and leaders from IBD patient associations were involved in the review of educational materials to ensure linguistic clarity, cultural relevance, and acceptability. They did not participate in data analysis or manuscript drafting.
